# 2951. Nursing home clinician characteristics associated with simulated urinary tract infection treatment decision-making

**DOI:** 10.1093/ofid/ofad500.190

**Published:** 2023-11-27

**Authors:** Lindsay Taylor, Brigid Wilson, Sally Jolles, Taissa A Bej, Corinne Kowal, Oteshia Hicks, Robin Jump, Christopher J Crnich

**Affiliations:** University of Wisconsin School of Medicine and Public Health, Madison, WI; VA Northeast Ohio Healthcare System, Cleveland, Ohio; University of Wisconsin School of Medicine and Public Health, Madison, WI; Louis Stokes Cleveland VA Medical Center, Cleveland, Ohio; Louis Stokes Cleveland VA Medical Center, Cleveland, Ohio; VA Northeast Ohio Healthcare System, Cleveland, Ohio; VA Pittsburgh Healthcare System, Fairview Park, OH; University of Wisconsin School of Medicine and Public Health, Madison, WI

## Abstract

**Background:**

Antibiotic overuse and misuse are common in nursing homes (NHs). Urinary tract infection (UTI) is the most common indication for antibiotics in NHs. We conducted a clinical vignette experiment to identify clinician-level characteristics associated with suboptimal UTI treatment decision-making among a sample of NH clinicians.

**Methods:**

Six clinical vignettes, two designed to assess clinicians’ treatment threshold and four designed to assess antibiotic choice decision-making, were created through an iterative process. NH clinicians were recruited nationally via professional organizations from December 2021 to April 2022. Clinical vignettes were shared using a web-based survey tool (Qualtrics) during which participants were asked if they would initiate antibiotics and which one. Participants provided demographic information, including degree, specialty training and NH experience. A multivariable logistic regression model with the decision to initiate antibiotic therapy (Yes/No) as the dependent variable and a multivariable ordinal regression model with number of vignettes for which a fluoroquinolone (FQ) was empirically chosen as the dependent variable were constructed in R.

**Results:**

A total of 298 NH clinicians, whose characteristics are detailed in **Table 1**, responded to the survey. Despite national guidelines not recommending treatment for asymptomatic bacteriuiria (ASB), 10.4% of participants chose to initiate antibiotics when presented with the ASB vignette. Physicians (*vs.* APPs) and clinicians with ≥ 10 years NH experience were less likely to initiate antibiotics for ASB (**Table 2**). Empiric treatment was withheld for simple cystitis by 38.9% of clinicians, with APPs more likely to withhold antibiotics than physicians (**Table 2**). Across the four cases assessing empiric antibiotic choice, 38.3% of clinicians chose a FQ in at least one clinical case (**Table 1**). Clinicians with more years of NH-practice experience were more likely to prescribe a FQ (aOR 2.41, 95% CI 1.47-3.95, p< 0.01, **Table 2**).

Respondent Characteristics

**Figure:**
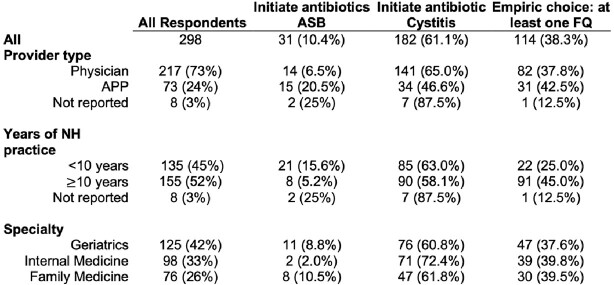

Abbreviations: Advanced Practice Provider (APP); Asymptomatic bacteriuria (ASB); Fluoroquinolone (FQ), Nursing Home (NH).

Effect of clinician credentials, training, and experience on antibiotic prescribing for urinary tract infection

**Figure:**
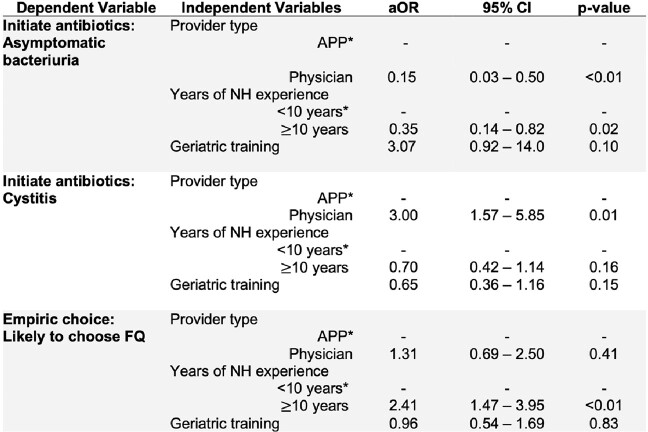

Abbreviations: * reference; Adjusted odds ratio (aOR); Confidence interval (CI); Asymptomatic bacteriuria (ASB); Advanced practitioner (APP)

**Conclusion:**

Our results indicate that specific clinician characteristics are associated with suboptimal antibiotic prescribing. This suggests that tailoring stewardship interventions to clinician-specific factors may augment ongoing efforts to improve antibiotic use in NHs.

**Disclosures:**

**Lindsay Taylor, MD, MS**, Merck: Grant/Research Support **Robin Jump, MD, PhD**, Merck: Grant/Research Support|Pfizer: Advisor/Consultant|Pfizer: Grant/Research Support

